# Does the Norwegian emergency medical dispatch classification as non-urgent predict no need for pre-hospital medical treatment? An observational study

**DOI:** 10.1186/s13049-016-0258-8

**Published:** 2016-05-06

**Authors:** Eystein Grusd, Jo Kramer-Johansen

**Affiliations:** Institute of Health and Society, Faculty of Medicine, University of Oslo, PO Box 1130, Blindern, 0318 Oslo Norway; Division of Prehospital Services, Ambulance Department, Oslo University Hospital HF, PO Box 4950, Nydalen, 0424 Oslo Norway; Norwegian National Advisory Unit on Prehospital Emergency Medicine (NAKOS), Oslo University Hospital and University of Oslo, Ullevål, PO Box 4950, Nydalen, 0424 Oslo Norway

**Keywords:** Ambulance service, Pre-hospital medical treatment, Need predictor, Acuity level, Norway, Emergency medical dispatch

## Abstract

**Background:**

The number of ambulance call-outs in Norway is increasing owing to societal changes and increased demand from the public. Together with improved but more expensive education of ambulance staff, this leads to increased costs and staffing shortages. We wanted to study whether the current dispatch triage tools could reliably identify patients who only required transport, and not pre-hospital medical care. This could allow selection of such patients for designated transport units, freeing up highly trained ambulance staff to attend patients in greater need.

**Methods:**

A cross-sectional observational study was used, drawing on all electronic and paper records in our ambulance service from four random days in 2012. The patients were classified into acuity groups, based on Emergency Medical Dispatch codes, and pre-hospital interventions were extracted from the Patient Report Forms.

**Results:**

Of the 1489 ambulance call-outs included in this study, 82 PRFs (5 %) were missing. A highly significant association was found between acuity group and recorded pre-hospital intervention (*p* ≤ 0.001). We found no correlation between gender, distance to hospital, age and pre-hospital interventions. Ambulances staffed by paramedics performed more interventions (234/917, 26 %) than those with emergency medical technicians (42/282, 15 %). The strongest predictor for needing pre-hospital interventions was found to be the emergency medical dispatch acuity descriptor.

**Discussion:**

This study has demonstrated that the Norwegian dispatch system is able to correctly identify patients who do not need pre-hospital interventions. Patients with a low acuity code had a very low level of pre-hospital interventions. Evaluation of adherence to protocol in the Emergency Medical Dispatch is not possible due to the inherent need for medical experience in the triage process.

**Conclusions:**

This study validates the Norwegian dispatch tool (Norwegian index) as a predictor of patients who do not need pre-hospital interventions.

## Background

 A large amount of health resources are used to treat and transport patients in the ambulance service Owing to an ageing population, sociological changes in society and increasing population size, the number of ambulance journeys and the cost of pre-hospital services are rising every year in Norway. In Norway health care is funded by the government, and total expenditure on health care in 2014 was 91 135 million NOK. Out of the total expenditure, ambulance services was 5 378 million NOK, while patient transports was 3 135 million NOK [1]. From 2010–2014 the total increase in health care expenditure was 6 %, while ambulance services increased 13,7 %. At the same time, pre-hospital care providers have to do more advanced diagnostics and treatment, increasing the costs of staff training and education still further. Current legislation and practice in Norway does not distinguish between patients in need of basic or advanced medical care and those who only require transport with a stretcher [2]. The authors have personally experienced on a daily basis the situation where ambulances staffed by paramedics transport patients who do not need any pre-hospital medical care while critically injured and sick patients are treated by less highly skilled staff. For the same reasons, a future shortage of skilled health personnel, including ambulance personnel, is likely. Transfer of low-acuity medical calls to transportation units could potentially save resources and maintain medical readiness in the system, but feasibility of such changes must be studied in our system to verify over-triage found in other systems [3, 4].

### Research questions

The main research question was:

- *What is the correlation between the emergency medical dispatch code of acuity and pre-hospital interventions performed on the patient as a marker of need for ambulance?*

We wanted to find out if a low acuity dispatch classification and the registered chief complaint could predict patients who were not in need of medical interventions in transit. We also explored patient attributes such as age, sex, and distance to hospital and formal competence of the ambulance staff as possible predictors of pre-hospital interventions performed on the patient.

## Methods

### EMS system

The ambulance service in the city of Oslo and surrounding county of Akershus is organized as a pre-hospital center under Oslo University Hospital Hospital Trust(HF). The ambulance service covers all types of ambulance transport in the area, both emergency (911) calls and interfacility transport. The service covers 5500 km^2^ across both urban and rural areas, and a population of 1,150,000. In total there are 48 ambulances and 15 ambulance stations staffed by emergency medical technicians (EMTs) and paramedics, who responded to 127,000 calls in 2011. Most ambulances are staffed with one EMT and one paramedic, or two EMTs. All ambulances have ALS capabilities. As required by law [2] EMT’s need licensure as EMT(Ambulansearbeider). To qualify, students need two years of vocational high school followed by two years of on the job training. Paramedics need licensure as “Ambulansearbeider” and a University College degree of 60 to 180 European Credit Transfer and Accumulation System points(ECTS). 60 ECTS points equals one year full time study at university level. Licenses are handled by The Norwegian Registration Authority for Health Personnel (SAK). By system design, all vehicles are ALS and can respond to all calls, but the emergency medical dispatch (EMD) operator may choose to differentiate assignments by acuity. All paramedics are able to carry out advanced pre-hospital procedures, such as administering medication, advanced airway procedures and intravenous cannulation. Some EMTs can also use advanced pre-hospital procedures, after a telephone consultation with a Medical Oversight Physician.

### Emergency medical dispatch system

**Fig. 1:**
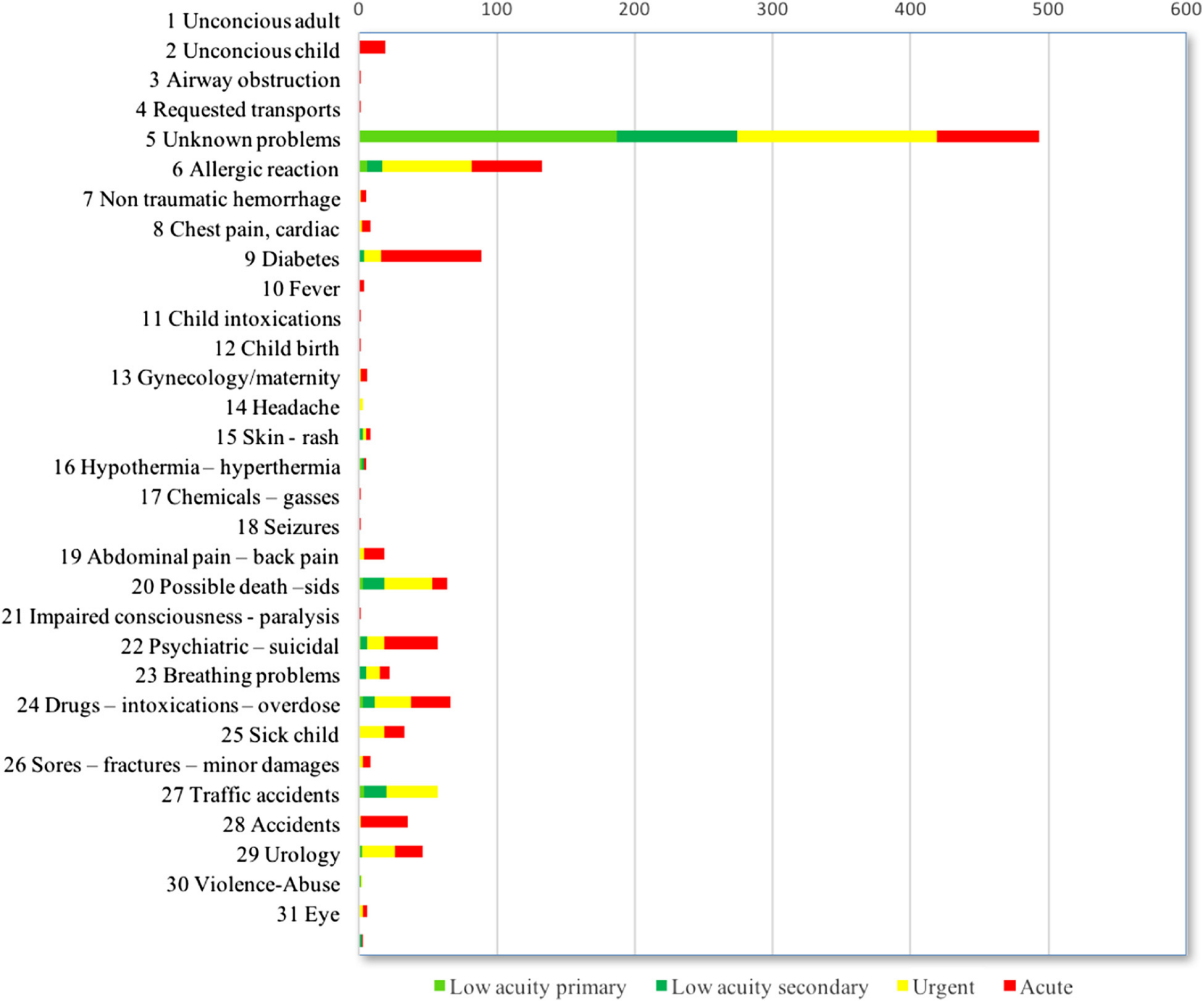
Distribution of acuity group within emergency medical dispatch code chief complaint classification

The EMD centers are staffed by registered nurses and paramedics who take calls from the general public, healthcare facilities and other cooperating partners such as the Police and the Fire Department. EMD operators are required by Norwegian law to be licensed health care workers, and undergo internal training at the EMD after employment. Although many EMD operators are cross trained, registered nurses mainly takes calls from the general public and triage patients while paramedics take calls from cooperating partners and dispatch ambulances. The EMD operators use a semi-structured approach in the telephone interview aided by the Norwegian index for medical dispatch [5]. Norwegian index is based on Criteria Based Dispatch [6], utilizing events, signs and symptoms to prioritize events into one of four acuity groups and one of 39 symptoms groups (see Tables 1 and 2 for details). The EMD operator categorized the calls into “low acuity”, “urgent” and “acute”, with increasing levels of acuity. Low acuity calls were further divided partially based on whether the patient was at home (primary) or in a healthcare facility (secondary). For the analysis, acuity groups were dichotomized to high (acute and urgent) and low acuity. The EMD operator also categorized each event into one of 39 symptom groups. Any symptom group with less than 2 % of the total call volume was excluded from the statistical analysis (see Fig. 1 for the symptom groups).

**Table 1:** Distribution of patients among the acuity groups

Acuity	All patients (*n* = 1200)	Pre-hospital interventions (*n* = 277)	No pre-hospital interventions (*n* = 923)	*p*-value
Acute (%)	424 (35.3 %)	174 (62.8 %)	250 (27.1 %)	
Urgent (%)	406 (33.8 %)	88 (31.8 %)	318 (34.5 %)	
Low acuity primary (%)	161 (13.4 %)	8 (2.9 %)	153 (16.6 %)	
Low acuity secondary (%)	209 (17.4 %)	7 (2.5 %)	202 (21.9 %)	<0.01

Description of acuity groups: *Acute:* Patient in need of immediate medical response due to imminent or potential loss of physological stability. *Urgent:* Patient in need of medical assessment without delay but with intact vital signs. *Low aquity primary:* Patient in need of transportation or assessment but supposedly stable. *Low aquity secondary:* Same as above, but transport requested from other medical presonnel (between institutions or to/from appointments)

**Table 2:** Distribution of patients across the main emergency medical dispatch codes, excluding oxygen as a pre-hospital intervention

Main emergency medical dispatch code	*n* = 1200	Pre-hospital interventions (*n* = 277)	No pre-hospital interventions (*n* = 923)	*p*-value
1 Unconscious adult	19 (1.6 %)	10 (3.6 %)	9 (1 %)	
2 Unconscious child	1 (.1 %)	0	1 (.1 %)	
3 Airway obstruction	1 (.1 %)	0	1 (.1 %)	
4 Requested transports	493 (41.2 %)	38 (13.7 %)	455 (49.5 %)	
5 Unknown problem	133 (11.1 %)	39 (14.1 %)	94 (10.2 %)	
6 Allergic reaction	5 (.4 %)	4 (1.4 %)	1 (.1 %)	
7 Non traumatic hemorrhage	8 (.7 %)	1 (.4 %)	8 (.8 %)	
8 Chest pain, cardiac	89 (7.4 %)	71 (25.6 %)	18 (2 %)	
9 Diabetes	4 (.3 %)	1 (.4 %)	1 (.3 %)	
10 Fever	1 (.1 %)	0	1 (.1 %)	
11 Child intoxications	1 (.1 %)	0	1 (.1 %)	
12 Child birth	6 (.5 %)	0	6 (.7 %)	
13 Gynecology/maternity	3 (.3 %)	0	3 (.3 %)	
14 Headache	8 (.7 %)	0	8 (.9 %)	
15 Skin - rash	5 (.4 %)	0	5 (.5 %)	
16 Hypothermia – hyperthermia	1 (.1 %)	0	1 (.1 %)	
17 Chemicals – gasses	1 (.1 %)	0	1 (.1 %)	
18 Seizures	18 (1.5 %)	5 (1.8 %)	13 (1.4 %	
19 Abdominal pain – back pain	64 (5.3 %)	19 (6.9 %)	45 (4.9 %)	
20 Possible death – sudden infant death	1 (.1 %)	1 (.4 %)	0	
21 Impaired consciousness - paralysis	57 (4.8 %)	19 (6.9 %)	38 (4.1 %)	
22 Psychiatric – suicidal	22 (1.8 %)	1 (.4 %)	21 (2.3 %)	
23 Breathing problems	66 (5.5 %)	29 (10.5 %)	37 (4 %)	
24 Drugs – intoxications – overdose	33 (2.8 %)	10 (3.6 %)	23 (2.5 %)	
25 Sick child	8 (.7 %)	2 (.7 %)	6 (.7 %)	
26 Sores – fractures – minor damages	57 (4.8 %)	13 (4.7 %)	44 (4.8 %)	
27 Traffic accidents	35 (2.9 %)	8 (2.9 %)	27 (2.9 %)	
28 Accidents	46 (3.8 %)	4 (1.4 %)	42 (4.6 %)	
29 Urology	2 (.2 %)	0	2 (.2 %)	
30 Violence – Abuse	6 (.5 %)	1 (.4 %)	5 (.5 %)	
31 Eye	3 (.3 %)	1 (.4 %)	2 (.2 %)	<0.001

**Table 3:** Combined model of attributes of patients who did not receive pre-hospital interventions with *p* ≤ 0.25 after performing backwise logistical regression, including demographic variables with *p* ≥ 0.25

	Beta	SE	*P*-value	OR (95 % CI)
Age in years	0.012	.004	.001	1.012 (1.005, 1.020)
Sex (male)	0.142	.167	.400	1.153 (.831, 1.600)
Low acuity primary				reference
Low acuity secondary	−.553	.378	.140	.575 (.274, 1.205)
Urgent	1.043	.265	<.001	2.839 (1.690, 4.770)
Acute	2.111	.276	<.001	8.259 (4.810, 14.179)
Chest pain, cardiac	2.059	.370	<.001	7.842 (3.796, 16.198)
Abdominal pain, back pain	.740	.333	.030	2.096 (1.090, 16.198)
Breathing problems	1.430	.345	<.001	4.178 (2.125, 8.214)
Accidents	−1.325	.628	.040	.266 (.078, .910)
Minutes used on transport	.020	.004	<.001	1.020 (1.013, 1.028)

### Patient documentation

All electronic records were extracted from the EMD dispatch database, and paper Patient Report Forms (PRF) were collected from the ambulance stations. The computer-aided dispatch system contains general information about the patient and call, including timestamps, demographic data, sex, birthdate, unit deployed, acuity level, chief complaint, any free text entered by the call-handler and an unique ID which can later be matched with the pre-hospital paper PRF. In the ambulance, a paper report is written which contains demographic data about the patient, a standard medical observation curve for vital signs, and details of any medication or treatment provided. There are free text fields for patient history, observations and treatment, and several scoring tools and checkbox lists, such as Glasgow Coma Scale and a trauma check list.

### Study setting and design

In this observational study, four separate days in September 2012 were randomly selected using Excel’s random function. The four days could be any week day during this month, and one week day could be selected more than once. From the EMD database, a comma separated value (csv) file was extracted, excluding names, addresses and birthdates. Interviews with EMTs and paramedics staffing the ambulances on the four study days were undertaken onsite directly after calls to validate the written patient reports. The interviews consisted of simple questions, addressing the use of pre-hospital interventions, and comparing the answers with the written patient report. One of the chosen random days was later changed to another, because there was a funeral on the same date, which led to practical problems conducting interviews onsite. A new day was selected utilizing Microsoft Excel random() function, and any day in the study period was selectable.

From the ambulance PRFs, information about pre-hospital procedures was extracted into a spreadsheet (Microsoft Excel for Mac 14.3.5). The information extracted was medication given, intravenous fluid therapy, advanced airway procedures, cardiopulmonary resuscitation, defibrillation, 12-lead electrocardiogram, intravenous cannulation, oxygen administration and the use of extrication and stabilization equipment. Available advanced airway procedures in this ambulance service are oropharyngeal airway, endotracheal intubation and a supraglottic device (“iGel”). Medications available are acetaminophen (paracetamol), acetylsalicylic acid (aspirin), amiodarone, clopidogrel, heparin, diazepam, epinephrine, furosemide, glucose 500 mg/ml, morphine, hydrocortisone, ipratropium bromide, metoclopramide, naloxone, nitroglycerin, and salbutamol.

Medical interventions performed on the patient were dichotomized according to whether there was any intervention. Because some patients may use oxygen permanently at home, or the facility from where the patient was collected may have administered oxygen prior to the ambulance arrival, use of oxygen was excluded from the interventions group classification. Since distance to hospital, and therefore travel time, may contribute to the assessment and interventions, a new variable was added as well as the timestamps. Based on the ambulance stations’ distance from the nearest hospital the events at this station were classified as “close to the hospital” if estimated travel time was less than 15 min, and “far from the hospital” for the rest of the ambulance stations.

### Statistical analysis

Continuous values were described as medians with interquartile ranges, owing to their non-normal distribution. Categorical variables were described as proportions within the group. Mann–Whitney and chi-square tests were used for comparisons, and *P*-values < 0.05 were regarded as significant. A logistic, backward, stepwise regression was performed to explore the relationship between pre-hospital factors and the need for interventions. We started with all variables with *P*-values < 0.25, then took out the variable with the highest *p*-value, until all variables had a *p*-value of < 0.05. All statistical analyses were performed using Statistical Package for Social Sciences (SPSS Mac©), version 21.0 (IBM Corp., Armonk, NY).

### Ethics

This project was an internal quality assurance project within the pre-hospital center of Oslo University Hospital HF, and the study was recommended by the Data Protection Official for research at Oslo University Hospital (2012/9274). The project was also considered by the Regional Committee for Medical and Health Research Ethics and found to be outside the scope of medical research. Since it was an internal quality assurance project, no consent form was required from patients or employees at Oslo University Hospital. However, an information leaflet was provided to employees before the interviews, explaining the project and making clear that they could decline to be interviewed. All employees who received the information leaflet chose to participate. All patient data were already collected within the hospital, and the researcher had no access to names, birthdates or other clearly-identifiable data from the computerized archive. PRFs were scanned with a prefabricated layover, which covered any demographic data such as names, addresses and birthdates. After data collection, all code lists were destroyed, and the resulting dataset was therefore completely anonymized.

## Results

In our 4-day study period, a total of 1489 ambulance journeys were dispatched by the EMD operators. Forty were excluded because they used resources without any patient transport capability, such as the ambulance supervisor, rapid response vehicle or a physician-staffed rapid response vehicle. Seventeen journeys were performed by voluntary agencies, and they were also excluded because records for them are archived outside the hospital. One hundred fifty of the recorded dispatches did not result in a patient encounter, and were also excluded. Eighty-two PRFs (5 %) were missing. Some cases belonged to more than one exclusion category. After exclusion criteria were applied, complete data were available for 1200 ambulance journeys. As expected, the study group age was not normally distributed, having a mean age of 59 (range 0–99) and median of 63. About one quarter, 282 (23 %), of the total ambulance journeys had only EMTs staffing the vehicle. The distribution by acuity categories and characteristics of the patients is set out in Table 1.

### Interviews

During the study period, 52 interviews were conducted onsite with the ambulance personnel, and the results were compared with the written patient report. In one of these interviews, one pre-hospital intervention was not documented correctly on the curve in the PRF but was written in the free text field instead. Finally 96.2 % of the pre-hospital interventions were correctly documented. Two cases in the high acuity group were found to be missing additional documentation such as Utstein [7] reports after cardiac arrest.

### Relationship between the EMD-allocated acuity level and chief complaint classification, and pre-hospital interventions

As expected, there was a highly significant association between acuity group and recorded pre-hospital intervention, *p* ≤ 0.001.

The majority of pre-hospital interventions were carried out on patients in just a few symptom groups. Most patients were classified as “requested transport” and this included request for ambulance from other health care personnel, police or fire dispatchers. The high acuity calls in this group typically included traffic incidents or other accidents. Eighty percent of patients with chest pain or cardiac symptoms received interventions (71/89), while none of the eight patients in the symptom group “headache” received any pre-hospital interventions. In the ‘unknown problem’ group, 30 % received some form of pre-hospital interventions, and in the low acuity secondary group, 8 % received a pre-hospital intervention.

### Effect of patient attributes such as age, sex and distance to hospital

The median age of those receiving interventions was the same as in the group that did not receive interventions (63 years, range 0–99). There was also no significant association between gender and pre-hospital interventions, with *p* = 0.46. The distance to hospital from the ambulance station was cross-tabulated and a chi-square test performed. There was no significant association with pre-hospital interventions (*p* = 0.39).

### Relationship between competency of the ambulance personnel and pre-hospital interventions

The proportion of ambulance journeys with interventions was higher for vehicles staffed by paramedics (234/917, 26 %) than those with EMTs (42/282, 15 %, *p* ≤ 0.001.

A backward stepwise logistical regression was performed on all attributes from the patient encounter, to assess a number of factors that might predict the need for pre-hospital interventions. The model contained seven independent variables; sex, age, distance from the hospital, travel time in minutes, time of day, competency level of the ambulance staff, and acuity and chief complaint groups from the EMD. The final model (Table 3) contained the predictors age, assigned acuity level, travel time and the symptom groups chest pain, breathing difficulties, abdominal pain and accidents, and was statistically significant, *χ*^2^ (5, *N* = 995) = 303.64, *p* ≤0.001, indicating that the final model distinguished between patients who did and did not receive pre-hospital intervention. The model explained between 26.3 % (Cox and Snell R square) and 40.1 % (Nagelkerke R square) of the variance in pre-hospital interventions. The final model correctly identified 95.1 % of the patients who did not receive pre-hospital interventions, and 36.9 % of the patients who did. The Hosmer and Lemeshow goodness of fit test on the final model with eight degrees of freedom gives a chi-square of 6.36 with *p* = 0.61, indicating a good fit for the model. The strongest predictor for pre-hospital intervention is the EMD acuity descriptor. When the level of need is acute, the odds ratio for any intervention is 8.2 (95 % CI:4.8–14.1) times greater than in the low acuity secondary group. Chest pain/cardiac problems were also a strong predictor with an odds ratio of 7.8 (95 % CI:3.7–16.2).

## Discussion

This study has demonstrated that the Norwegian ambulance dispatch system is able to correctly identify patients who do not need pre-hospital interventions, with a considerable degree of overtriage for the group receiving pre-hospital intervention. This is similar to previous research done on other call handler triage systems [3, 4]. If the triage system is effective in identifying patients who do not need pre-hospital interventions, the level of interventions should decline from the highest acuity group to the lowest. In this study, the two high acuity groups had a significantly higher level of pre-hospital interventions than the two lower groups. The symptom groups of chest pain/cardiac problems, breathing difficulties and abdominal pain all have a higher proportion of pre-hospital interventions. Patients in other symptom groups and with a low acuity code had a very low level of pre-hospital interventions. The level of training (EMT vs. paramedic) influenced on the proportion of patients receiving prehospital interventions, but this relationship disappeared in the regression analysis, probably due to selection of ambulances resources to different calls based on acuity.

The EMD operator process using Norwegian index differs from the rigidly structured questions in Medical Priority Dispatch systems, and necessitates medical qualifications, that also allows operators to bypass questions if callers explain symptoms sufficiently without prompting [8]. In addition to all Norwegian EMD centers, Swedish and Danish EMD centers use translated versions of the same Criteria Based Dispatch protocol [9]. Internal quality assurance includes audit of selected calls by supervisors, but a rigid evaluation of adherence to protocol is not possible due to the inherent need for medical experience in the triage process, especially for the low-acuity calls.

### Interviews

Staff were interviewed in the selected cases, and most journals were accurate, although some inconsistencies were found. A high degree of missing PRFs was consistent with previous research on Norwegian pre-hospital paper records [10]. Using electronic journals has been found to enhance research, accuracy, readability and consistency in hospital patient records [11, 12]. Technology exists to provide pre-hospital care providers with electronic journals, and this has been implemented in many ambulance services around the world [13, 14]. If this ambulance service had been using electronic patient records, the dataset would have had fewer missing records, and could have been easier to analyze.

### Acuity/chief complaint

Using low acuity as the sole predictor for no pre-hospital intervention showed a high degree of sensitivity, and could reliably be used to predict patients who do not need pre-hospital treatment. However the specificity of the dispatch protocol is low, and there is a high degree of overtriage. It could be argued that owing to the rising cost of healthcare and the general demographic move towards an older and sicker population, ambulances staffed by paramedics should be sent only to those patients who need pre-hospital assessment and treatment.

These findings can be used to separate patients with a lower need for pre-hospital interventions, and who could use transport resources staffed by staff with a lower level of competence. This would enable more cost-effective use of resources. In future, a new “lower competency” level of ambulance transport can safely transport patients who fit these criteria. However, the desired level of sensitivity has to consider both political and economic issues, which is outside the scope of this article.

We also suggest that all healthcare facilities, including pre-hospital providers, should adopt electronic patient records. This would make quality assurance and research projects easier. Adherence to treatment protocols could also be monitored easily and systematically, which could lead to improved patient safety.

### Limitations

This study had some limitations. First, just because a pre-hospital intervention was performed does not mean that it was absolutely necessary and vice versa. The interviews were only done during daytime, 08.00–18.00. Utilizing historic data from this EMS service, it was known that the majority of patient transports in this system were performed between 08.00 and 18.00. Interviews were then conducted onsite within this time frame. Additionally, the model does not include the need for qualified monitoring, with the possibility of pre-hospital interventions. As Sporer [15] pointed out, the use of pre-hospital interventions such as administering medication can be used as a proxy for the underlying need for qualified pre-hospital assessment, owing to the lack of a standardized definition for advanced pre-hospital assessment. The missing PRFs also limit the statistical validity of this study. However, these journals had the same distribution within the acuity groups as those included, so it is likely that the effects of this loss were small.

## Conclusions

Ninety-eight percent of the patients in the two low-acuity groups did not receive any pre-hospital interventions. A combined model of symptom group and acuity level recognized 95 % of this group. This validates the Norwegian index’s ability to predict those patients who do not need immediate medical treatment. More research is necessary on other aspects of the Norwegian dispatch protocol, to ensure patients receive the required level of healthcare and transport services.

## Abbreviations

ALS: advanced life support
BLS: basic life support
ECTS: European Credit Transfer and Accumulation System
EMD: emergency medical dispatch
EMT: emergency medical technician
HF: hospital trust
PRF: patient report form
SAK: The Norwegian Registration Authority for Health Personnel
